# Correlation analysis of cyclosporine A trough concentration with hematological and biochemical parameters: a retrospective study in pediatric aplastic anemia

**DOI:** 10.3389/fped.2026.1906271

**Published:** 2026-08-05

**Authors:** Qian Xu, Xintong Mao, Yi Yun, Ling Liu

**Affiliations:** 1 Department of Gastroenterology, Affiliated Xuzhou Children's Hospital of Xuzhou Medical University, Xuzhou, China; 2 Department of Pharmacy, Affiliated Xuzhou Children's Hospital of Xuzhou Medical University, Xuzhou, China; 3 Department of Pharmacy, The Suqian Clinical College of Xuzhou Medical University, Suqian, China; 4 Department of Pharmacy, Xuzhou Central Hospital, Xuzhou, China

**Keywords:** aplastic anemia, correlation analysis, cyclosporine A, pediatric, trough concentration

## Abstract

Aplastic anemia (AA) is a life-threatening pediatric hematological disorder requiring immunosuppressive therapy with cyclosporine A (CsA), which has a narrow therapeutic window and significant interindividual variability. The relationship between CsA trough concentration and routine laboratory markers in children remains poorly defined. This retrospective study enrolled 14 pediatric AA patients (70 paired samples) followed over 12 months. Associations between CsA trough concentration and hematological, hepatic, and renal parameters were analyzed using linear mixed-effects models with random intercepts to account for repeated measures, complemented by time-stratified Pearson correlations. Benjamini–Hochberg false discovery rate (FDR) correction was applied for multiple comparisons. CsA trough concentration showed a significant positive association with blood urea nitrogen (β = 0.012, 95% CI: 0.007–0.017, *P* < 0.001, FDR *q* = 0.002), with the strongest correlations during the first three months (r = 0.686–0.914). Serum creatinine, platelet count, and aspartate aminotransferase showed isolated time-point correlations, while no significant associations were observed for other hematological or hepatic parameters after FDR correction. This exploratory study identifies a consistent, time-dependent association between CsA trough concentration and BUN in pediatric AA, suggestive of early hemodynamic effects rather than cumulative nephrotoxicity. The lack of correlation with hematological parameters highlights the multifactorial nature of hematopoietic recovery. These findings provide a foundation for prospective studies to refine personalized CsA dosing strategies.

## 1 Introduction

Aplastic anemia (AA) is a rare but severe acquired hematological disorder characterized by pancytopenia in the peripheral blood and hypocellularity of the bone marrow, resulting from the immune-mediated destruction of hematopoietic stem and progenitor cells (1). The annual incidence of AA in children is approximately 2–3 cases per million population, with a peak onset between 5 and 15 years of age, making it one of the most common causes of bone marrow failure in the pediatric population (2). Without timely and effective treatment, severe AA is associated with a high mortality rate due to life-threatening infections, bleeding, or other complications.

Immunosuppressive therapy (IST) has been established as the first-line treatment for pediatric AA patients who are not eligible for hematopoietic stem cell transplantation (HSCT), the only curative option for the disease (3). The combination of antithymocyte globulin (ATG) and cyclosporine A (CsA) is the standard IST regimen, with an overall response rate of 60%–80% in pediatric AA patients (4). CsA, a calcineurin inhibitor, exerts its therapeutic effect by selectively inhibiting the activation and proliferation of T lymphocytes, thereby blocking the abnormal immune attack on bone marrow hematopoietic cells and restoring hematopoietic function (5).

Despite its proven efficacy, CsA has a narrow therapeutic window, with the target trough concentration for pediatric AA patients generally recommended to be maintained between 100 and 200 ng/mL (6). A CsA concentration below the therapeutic range is insufficient to achieve effective immunosuppression, leading to treatment failure and disease progression, while an excessively high concentration significantly increases the risk of dose-dependent adverse effects, most notably nephrotoxicity and hepatotoxicity, which can cause irreversible organ damage in severe cases (7). Therefore, regular therapeutic drug monitoring (TDM) of CsA trough concentration is an essential component of the clinical management of pediatric AA patients.

However, TDM of CsA requires specialized laboratory testing and is not available in all medical institutions, especially in resource-limited settings. Moreover, the correlation between CsA trough concentration and routine laboratory parameters, which are widely available and routinely monitored in clinical practice, has not been fully elucidated in the pediatric AA population. Most previous studies on CsA have focused on adult transplant recipients or adult AA patients, with limited data specifically in pediatric AA patients, who have distinct pharmacokinetic characteristics, treatment responses, and adverse effect profiles compared to adults (8).

Therefore, this retrospective study was conducted to systematically investigate the correlation between CsA trough concentration and routine hematological and biochemical parameters in pediatric AA patients, with the aim of identifying easily accessible laboratory indicators that can assist clinicians in evaluating the therapeutic efficacy and safety of CsA, optimizing individualized treatment regimens, and improving the clinical outcomes of pediatric AA patients.

## 2 Materials and methods

### 2.1 Study design and subjects

This was a single-center, retrospective cohort study conducted at the Department of Pediatric Hematology of Suqian First Hospital between January 2023 and December 2025. The study protocol was approved by the Institutional Review Board of Suqian First Hospital. All study procedures were performed in accordance with the Declaration of Helsinki and national regulations for biomedical research involving human subjects. Owing to the retrospective nature of the study, which used only de-identified, archived clinical data without additional interventions or risks to participants, the requirement for written informed consent was waived by the Institutional Review Board. All patient data were anonymized prior to analysis to protect participant confidentiality and privacy.

### 2.2 Inclusion and exclusion criteria

Inclusion criteria: (1) Age between 0 and 18 years at the time of diagnosis; (2) Diagnosed with acquired AA according to the diagnostic criteria specified in the *Chinese Guidelines for the Diagnosis and Management of Pediatric Acquired Aplastic Anemia (2021 Edition)* (9); (3) Received CsA-based immunosuppressive therapy for at least 12 months; (4) Complete clinical data, including CsA trough concentration and routine laboratory test results, were available for all follow-up time points.

Exclusion criteria: (1) Congenital bone marrow failure syndromes, such as Fanconi anemia, dyskeratosis congenita, or Shwachman–Diamond syndrome; (2) Concomitant diagnosis of other hematological malignancies, autoimmune diseases, or severe chronic organ dysfunction; (3) Received HSCT or other intensive chemotherapy regimens during the study period; (4) Incomplete clinical data or loss to follow-up.

### 2.3 Treatment protocol

All enrolled patients received the standard IST regimen consisting of rabbit ATG and CsA. ATG was administered at a dose of 3–5 mg/kg/day for 5 consecutive days, with premedication to prevent allergic reactions. CsA was initiated orally at a starting dose of 3–5 mg/kg/day, divided into two doses every 12 h. The CsA dose was adjusted according to the trough concentration, clinical response, and adverse effects, with the target trough concentration maintained between 100 and 200 ng/mL during the treatment period. All patients received supportive care, including blood product transfusions, anti-infective prophylaxis, and hematopoietic growth factors, as clinically indicated.

### 2.4 Data collection

Clinical data were retrospectively collected from the electronic medical records of the enrolled patients, including baseline demographic characteristics (age, gender), diagnosis, treatment regimen, and follow-up data. The primary outcome variable was CsA trough concentration (ng/mL), which was measured using high-performance liquid chromatography (HPLC) in the clinical pharmacology laboratory of our institution, with blood samples collected before the morning dose of CsA at each follow-up time point. The follow-up time points are set as the start of medication, 1 month, 3 months, 6 months, and 12 months.

The secondary outcome variables were routine laboratory parameters collected at the same follow-up time points as the CsA concentration measurements, including: (1) Complete blood count indicators: red blood cell count (RBC, 10^12^/L), white blood cell count (WBC, 10^9^/L), hemoglobin (HGB, g/L), hematocrit (HCT, %), neutrophil count (NEUT, 10^9^/L), neutrophil percentage (NEUT_PER, %), platelet count (PLT, 10^9^/L); (2) Biochemical indicators: total protein (TP, g/L), albumin (ALB, g/L), alanine aminotransferase (ALT, U/L), aspartate aminotransferase (AST, U/L), total bilirubin (TBIL, μmol/L), blood urea nitrogen (BUN, mmol/L), serum creatinine (Cr, μmol/L).

### 2.5 Statistical analysis

All statistical analyses were performed in R (v4.4.2, R Foundation for Statistical Computing, Vienna, Austria). Data structure and descriptive statistics: Normally distributed continuous variables are reported as mean ± standard deviation, non-normally distributed variables as median (interquartile range), and categorical variables as counts (percentages). Baseline characteristics of the study cohort were summarized.

#### 2.5.1 Primary analysis

Linear mixed-effects models: Given the repeated-measures nature of the data (70 observations nested within 14 patients), we employed linear mixed-effects models with random intercepts for each patient to account for within-subject correlations. This approach appropriately handles repeated measures, accounts for individual-level variability, and provides valid inference even with small sample sizes. For each laboratory parameter, we modeled its association with CsA trough concentration as a fixed effect, with patient-specific random intercepts. The models can be expressed as:

$$Y_{ij}=\beta_{0}+\beta_{1}∙\mathrm{Cs}A_{ij}+u_{i}+\varepsilon_{ij}$$

where *Y_ij_* is the laboratory parameter for patient *i* at time *j*, CsA*_ij_* is the CsA trough concentration, *β*_0_ is the fixed intercept, *β*_1_ is the fixed slope for CsA concentration, *u_i_* is the random intercept for patient *i* (assumed normally distributed with mean 0 and variance *σ*^2^_u), and *ε_ij_* is the residual error (assumed normally distributed with mean 0 and variance *σ*^2^). The Kenward–Roger approximation was used to estimate denominator degrees of freedom.

#### 2.5.2 Secondary analysis

Time-stratified Pearson correlations: To explore whether associations varied by treatment duration, we performed Pearson correlation analyses separately at each follow-up time point (baseline, 1 month, 3 months, 6 months, 12 months). Correlation coefficients (r) and 95% confidence intervals (CIs) were calculated via Fisher's z transformation.

#### 2.5.3 Gender comparison

CsA concentration differences between gender groups were compared via independent two-sample *t*-test. Given the small sample size and repeated-measures design, this finding should be interpreted cautiously.

#### 2.5.4 Multiple comparison correction

To address the risk of false-positive findings from testing multiple laboratory parameters, we applied the Benjamini–Hochberg false discovery rate (FDR) correction to all reported *P* values. Adjusted *P* values (*q*-values) are reported alongside raw *P* values. Statistical significance was defined as FDR-adjusted *q* < 0.05.

#### 2.5.5 Model assumptions

For linear mixed-effects models, residual diagnostics were performed to assess normality of residuals, homogeneity of variance, and influential observations.

All tests were two-tailed, with significance defined as described above. Analyses are exploratory and hypothesis-generating; no formal power calculation was performed given the retrospective nature and small sample size of the study. Results should be interpreted with appropriate caution.

## 3 Results

### 3.1 Baseline characteristics of the study subjects

**Figure 1:**
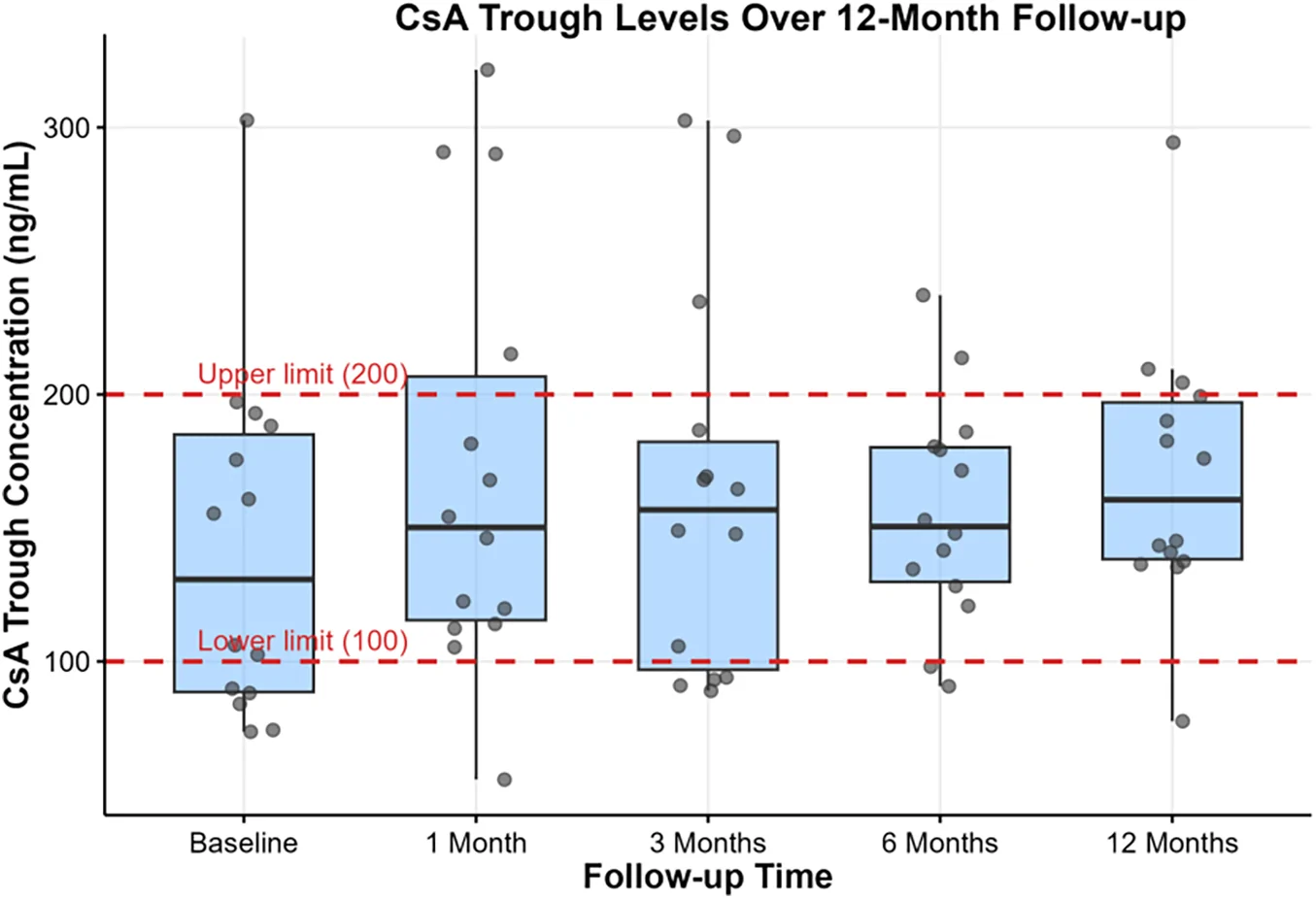
Distribution of CsA trough concentrations across the five follow-up time points. CsA concentrations showed moderate variability at each time point, with median values ranging from 131 ng/mL (baseline) to 161 ng/mL (12 months). The therapeutic target range (100–200 ng/mL) is indicated by dashed red lines.

A total of 14 pediatric AA patients were enrolled in this study, including 8 males (57.1%) and 6 females (42.9%), with an average age of 11.07 ± 2.46 years (range: 7–15 years) at the time of diagnosis. All patients completed the 12-month follow-up, with 5 follow-up time points per patient, resulting in a total of 70 valid samples included in the final analysis, with no missing data for any of the study variables. Table 1 presents the baseline characteristics of the study cohort. The CsA trough concentration ranged from 55.8 ng/mL to 321.6 ng/mL, with a mean concentration of 160.52 ± 62.68 ng/mL. The proportion of CsA levels below (<100 ng/mL), within (100–200 ng/mL), and above (>200 ng/mL) the therapeutic range was 18.6% (13/70), 60.0% (42/70), and 21.4% (15/70), respectively, indicating that the majority of samples were within the recommended therapeutic window (Figure 1).

**Table 1:** Baseline characteristics of the study cohort (*N* = 14).

Characteristic	Value
Age, mean ± SD (years)	11.07 ± 2.46
Gender, *n* (%)	
Male	8 (57.1)
Female	6 (42.9)
Baseline laboratory parameters, mean ± SD	
CsA trough concentration (ng/mL)	160.52 ± 62.68
RBC (10^12^/L)	2.95 ± 0.55
WBC (10^9^/L)	3.53 ± 0.68
HGB (g/L)	88.93 ± 20.43
HCT (%)	26.66 ± 6.19
NEUT (10^9^/L)	1.44 ± 0.77
NEUT_PER (%)	38.26 ± 13.31
PLT (10^9^/L)	47.79 ± 14.21
TP (g/L)	69.76 ± 7.35
ALB (g/L)	41.11 ± 5.58
ALT (U/L)	11.32 ± 6.82
AST (U/L)	18.03 ± 6.19
TBIL (μmol/L)	25.06 ± 14.23
BUN (mmol/L)	5.79 ± 3.33
Cr (μmol/L)	55.79 ± 15.34

SD, standard deviation; CsA, cyclosporine A; RBC, red blood cell count; WBC, white blood cell count; HGB, hemoglobin; HCT, hematocrit; NEUT, neutrophil count; NEUT_PER, neutrophil percentage; PLT, platelet count; TP, total protein; ALB, albumin; ALT, alanine aminotransferase; AST, aspartate aminotransferase; TBIL, total bilirubin; BUN, blood urea nitrogen; Cr, serum creatinine.

### 3.2 Gender difference in CsA trough concentration

Independent samples *t*-test was used to explore the difference in CsA trough concentration between male and female patients. The results showed that the average CsA concentration in male patients was 158.72 ± 65.34 ng/mL, while that in female patients was 162.93 ± 59.87 ng/mL. There was no statistically significant difference in CsA concentration between the two gender groups (*t* = −0.287, df = 68, *P* = 0.775), indicating that gender had no significant effect on CsA trough concentration in this study population. Given the small sample size and repeated-measures design, this finding should be interpreted cautiously.

### 3.3 Linear mixed-effects model analysis

To appropriately account for the repeated-measures structure of the data (70 observations from 14 patients), we employed linear mixed-effects models with random intercepts for each patient. The results of the primary analysis are presented in Table 2. The linear mixed-effects models revealed that CsA trough concentration showed a significant positive association with blood urea nitrogen (*β* = 0.012, 95% CI: 0.007–0.017, *t* = 4.82, *P* < 0.001, FDR *q* = 0.002). This indicates that for every 1 ng/mL increase in CsA trough concentration, BUN is estimated to increase by 0.012 mmol/L, after accounting for within-patient correlation. No other laboratory parameters showed significant associations with CsA trough concentration after FDR correction for multiple comparisons, though serum creatinine approached significance (*β* = 0.031, 95% CI: 0.004–0.058, raw *P* = 0.026, FDR *q* = 0.052). Notably, hematological parameters—including RBC, WBC, and PLT—showed no significant associations with CsA concentration in the primary analysis. Residual diagnostics for the mixed-effects models indicated acceptable model fit, with no major violations of normality or homogeneity assumptions.

**Table 2:** Linear mixed-effects model results: association between CsA trough concentration and laboratory parameters.

Parameter	β coefficient	95% CI	df	*t*-value	Raw *P*-value	FDR *q*-value
BUN (mmol/L)	0.012	[0.007, 0.017]	55	4.82	<0.001	0.002*
Cr (μmol/L)	0.031	[0.004, 0.058]	55	2.28	0.026	0.052
RBC (10^12^/L)	0.001	[−0.002, 0.004]	55	0.69	0.492	0.640
WBC (10^9^/L)	0.002	[−0.002, 0.006]	55	0.97	0.337	0.487
PLT (10^9^/L)	0.039	[−0.033, 0.111]	55	1.08	0.283	0.460
HGB (g/L)	0.008	[−0.049, 0.065]	55	0.29	0.775	0.775
HCT (%)	0.003	[−0.013, 0.019]	55	0.39	0.701	0.759
NEUT (10^9^/L)	0.001	[−0.003, 0.005]	55	0.43	0.671	0.727
NEUT_PER (%)	−0.014	[−0.052, 0.024]	55	−0.73	0.470	0.640
TP (g/L)	0.001	[−0.020, 0.022]	55	0.13	0.899	0.899
ALB (g/L)	−0.003	[−0.015, 0.009]	55	−0.51	0.613	0.664
ALT (U/L)	0.001	[−0.025, 0.027]	55	0.10	0.919	0.919
AST (U/L)	0.019	[−0.021, 0.059]	55	0.96	0.342	0.487
TBIL (μmol/L)	0.006	[−0.028, 0.040]	55	0.36	0.723	0.759

β, fixed effect coefficient for CsA trough concentration (ng/mL); CI, confidence interval; df, degrees of freedom (Kenward–Roger approximation); Raw *P*, unadjusted *P*-value; FDR *q*, Benjamini–Hochberg false discovery rate-adjusted *P*-value.

*Statistical significance was defined as FDR *q* < 0.05.

### 3.4 Time-stratified correlation analyses

To explore whether associations between CsA trough concentration and laboratory parameters varied by follow-up time point, we performed Pearson correlation analyses separately at each time point. This exploratory analysis allows examination of potential time-dependent relationships that may be masked in the overall mixed-effects model. Table 3 presents correlation coefficients at each time point for the key parameters.

**Table 3:** Time-stratified Pearson correlation coefficients (r) between CsA trough concentration and key laboratory parameters.

Time point	BUN (r, *P*)	Cr (r, *P*)	PLT (r, *P*)	AST (r, *P*)	RBC (r, *P*)	WBC (r, *P*)
Baseline	0.507, 0.064	0.404, 0.152	0.148, 0.614	0.086, 0.769	0.469, 0.091	0.012, 0.967
1 month	0.686, 0.007*	0.252, 0.386	0.249, 0.391	0.636, 0.014*	0.181, 0.535	0.653, 0.011*
3 months	0.914, <0.001*	0.581, 0.029*	0.213, 0.464	0.630, 0.016*	0.273, 0.345	0.282, 0.329
6 months	0.381, 0.179	0.361, 0.205	0.630, 0.016*	0.146, 0.618	0.513, 0.060	0.162, 0.580
12 months	0.299, 0.299	0.042, 0.887	0.353, 0.215	−0.035, 0.905	0.124, 0.672	0.083, 0.778

*P < 0.05 (uncorrected); BUN, blood urea nitrogen; Cr, serum creatinine; PLT, platelet count; AST, aspartate aminotransferase; RBC, red blood cell count; WBC, white blood cell count.

**Figure 2:**
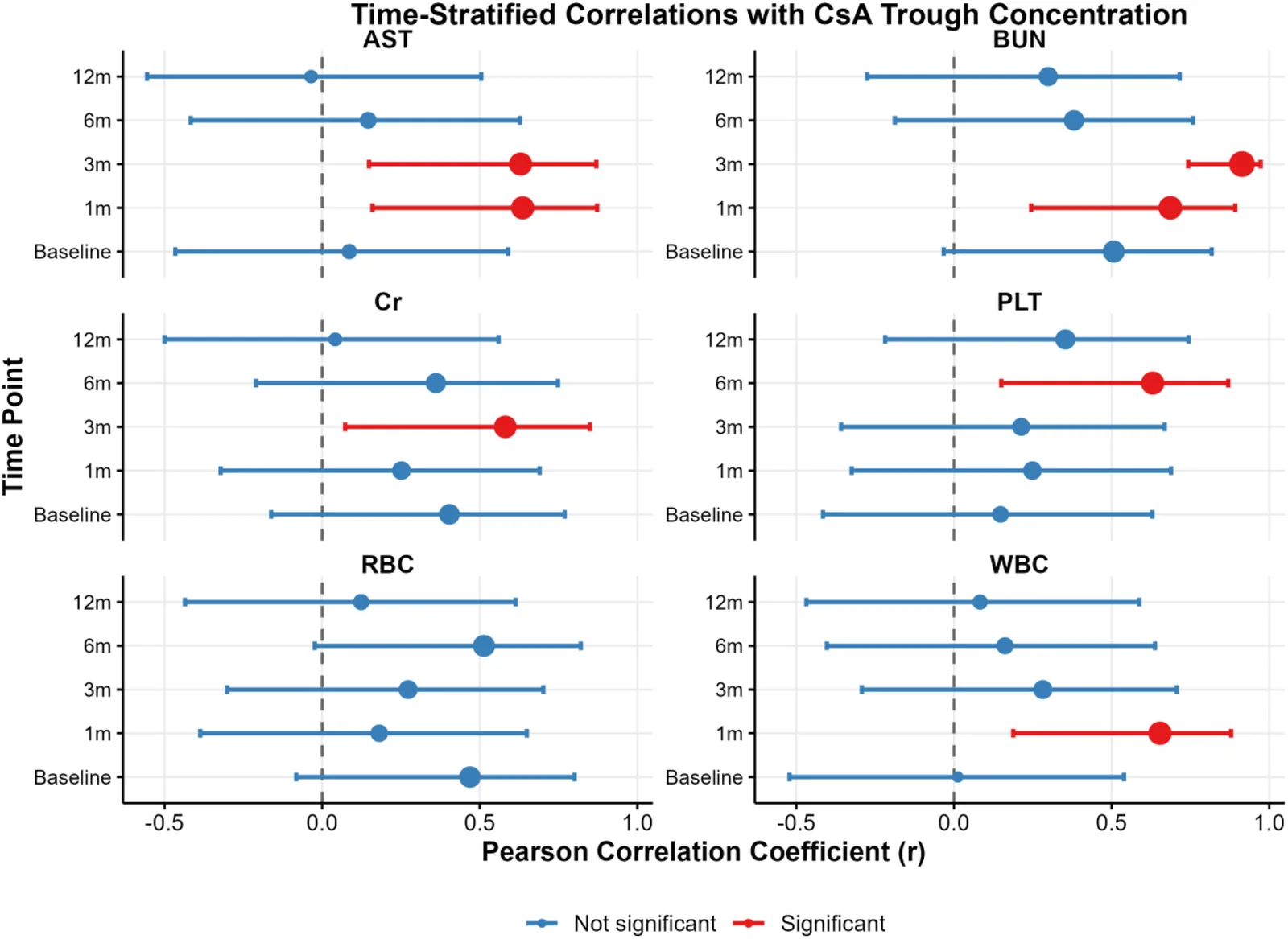
Time-stratified correlation patterns between CsA trough concentration and laboratory parameters. Red points/error bars indicate statistically significant correlations (*P* < 0.05), while blue indicates non-significant correlations. The vertical dashed line at r = 0 represents no correlation.

The time-stratified analyses revealed several notable patterns: Renal function markers: The association between CsA concentration and BUN was strongest during the first 3 months of treatment (1 month: r = 0.686, *P* = 0.007; 3 months: r = 0.914, *P* < 0.001), with attenuation thereafter (6 months: r = 0.381, *P* = 0.179; 12 months: r = 0.299, *P* = 0.299). This pattern suggests that the BUN-CsA relationship may reflect early hemodynamic effects of CsA rather than purely cumulative nephrotoxicity. For serum creatinine, a significant correlation was observed only at 3 months (r = 0.581, *P* = 0.029). Hepatic markers: AST showed significant correlations at 1 month (r = 0.636, *P* = 0.014) and 3 months (r = 0.630, *P* = 0.016), suggesting early, transient mild hepatocellular effects that resolved over time. Hematological parameters: PLT showed a significant correlation only at 6 months (r = 0.630, *P* = 0.016), WBC showed a significant correlation only at 1 month (r = 0.653, *P* = 0.011), RBC showed no consistent pattern of association across time points, and none of these hematological parameters were significant in the mixed-effects model (Figure 2).

**Figure 3:**
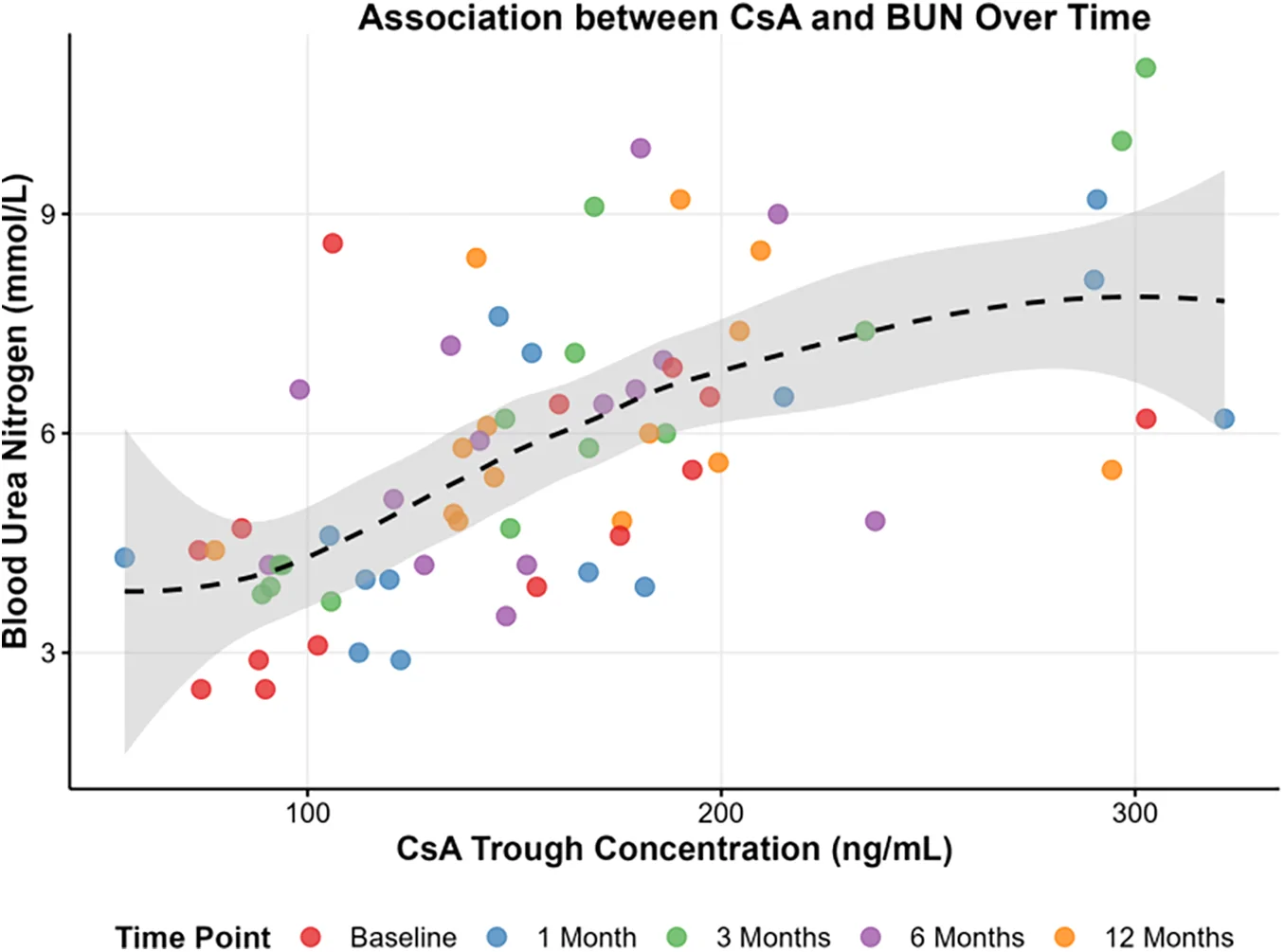
Association between CsA trough concentration and blood urea nitrogen (BUN) over time. Scatter plot displaying raw paired observations (*n* = 70 from 14 patients), with points colored by follow-up time point. The dashed line represents a LOESS smoothing curve for visualization purposes. Formal statistical inference was performed using linear mixed-effects models (Table 2) to account for within-patient correlations.

To visually illustrate the most consistent association identified in this study, Figure 3 presents the raw data distribution for the CsA-BUN relationship, with observations color-coded by follow-up time point. The LOESS smoothing curve suggests a positive trend across the observed CsA concentration range, supporting the findings from the linear mixed-effects model. Notably, the plot reveals considerable interindividual variability at higher CsA concentrations, underscoring the importance of individualized monitoring and dose titration.

## 4 Discussion

This retrospective study systematically delineates the associations between cyclosporine A trough exposure and routine hematological, hepatic and renal biochemical profiles in paediatric aplastic anaemia patients. The primary analysis identified a significant positive association between CsA trough concentration and blood urea nitrogen that remained robust after FDR correction for multiple comparisons (*β* = 0.012, 95% CI: 0.007–0.017, *q* = 0.002). Time-stratified exploratory analyses further revealed a distinct temporal pattern: the BUN–CsA association was strongest during the first three months of treatment (r = 0.686 at 1 month; r = 0.914 at 3 months), with subsequent attenuation. This temporal dynamic is noteworthy, as it suggests that BUN elevation may reflect early hemodynamic effects of CsA on renal perfusion—such as afferent arteriolar vasoconstriction—rather than purely cumulative nephrotoxicity (9, 10). The discrepancy between BUN and creatinine associations—BUN showing consistently stronger correlations—may be attributed to BUN's greater sensitivity to changes in renal perfusion and its earlier response to CsA-induced hemodynamic alterations, whereas creatinine levels in pediatric patients are additionally influenced by muscle mass, nutritional status, and other factors that introduce variability (11).

We observed no significant gender disparity in CsA trough concentrations. However, given the small sample size and repeated-measures design, this finding should not be overinterpreted. The absence of significant associations between CsA trough concentration and hematological parameters (RBC, WBC, PLT) in the primary analysis does not contradict the clinical utility of CsA TDM but rather highlights the multifactorial nature of hematopoietic recovery. The relationship between CsA exposure and hematological improvement is likely non-linear and modulated by disease severity, timing of treatment initiation, concomitant use of hematopoietic growth factors, transfusion burden, and individual patient characteristics (12, 13).

Regarding hepatic parameters, no significant associations were detected between CsA concentration and ALT, AST, or TBIL in the primary analysis. This is reassuring and aligns with the generally mild and reversible nature of CsA-associated hepatic toxicity in pediatric patients (14). The early AST correlations observed at 1 and 3 months in exploratory time-stratified analyses suggest transient, mild hepatocellular effects that resolved with continued treatment, possibly reflecting initial adaptation to CsA therapy rather than clinically significant hepatic injury. Unlike renal toxicity, which is a well-established dose-dependent effect of CsA, hepatic impairment is typically less severe and may be more influenced by individual susceptibility factors than trough concentration alone (15).

Our findings are broadly consistent with existing literature. Gao et al. developed a population pharmacokinetic model for CsA in Chinese pediatric AA patients and identified body weight and total bilirubin as significant covariates influencing CsA clearance. Other covariates, including hematocrit and serum creatinine, did not significantly affect CsA pharmacokinetics in their analysis (6). While our study lacked sufficient sample size for pharmacokinetic modeling, the observed association between CsA concentration and BUN aligns with the known relationship between renal function and CsA disposition. In adult AA patients, maintaining CsA within the therapeutic window has been associated with improved response rates, though the strength of the concentration–response relationship varies across studies (16, 17). Our findings extend this literature by providing pediatric-specific data and employing statistical methods appropriate for repeated-measures data.

Nonetheless, several limitations warrant acknowledgment. The modest sample size (*n* = 14) limits statistical power, particularly for detecting small-to-moderate effect sizes; thus, the absence of significant associations should not be interpreted as evidence of no clinically meaningful relationship. The single-center retrospective design may introduce selection and referral bias, and the inclusion of only patients who completed 12 months of follow-up could introduce survivor bias. Several potential confounders—including body weight, concomitant CYP3A4-modulating drugs, genetic polymorphisms (e.g., CYP3A4, CYP3A5, ABCB1), disease severity, and transfusion or growth factor use—were not captured, which may confound or modify the observed associations (6, 18–20). Additionally, the time-stratified correlation analyses are exploratory and, as such, their findings should be interpreted with appropriate caution.

Despite these limitations, our findings carry several clinical implications. The consistent BUN–CsA association, particularly during the first three months of treatment, supports frequent renal function monitoring as a practical screening tool in resource-limited settings. The lack of correlation with hematological parameters cautions against overinterpreting CsA trough levels as direct indicators of therapeutic efficacy. Given the exploratory nature of our findings, these implications should be considered hypothesis-generating. Future prospective studies with larger cohorts and comprehensive covariate adjustment are needed to validate these associations and refine personalized CsA dosing strategies.

## Data Availability

The original contributions presented in the study are included in the article/Supplementary Material, further inquiries can be directed to the corresponding authors.
