# Correction: Correlation analysis of cyclosporine a trough concentration with hematological and biochemical parameters: a retrospective study in pediatric aplastic anemia

**DOI:** 10.3389/fped.2026.1960380

**Published:** 2026-08-26

**Authors:** Qian Xu, Xintong Mao, Yi Yun, Ling Liu

**Affiliations:** 1 Department of Gastroenterology, Affiliated Xuzhou Children's Hospital of Xuzhou Medical University, Xuzhou, China; 2 Department of Pharmacy, Affiliated Xuzhou Children's Hospital of Xuzhou Medical University, Xuzhou, China; 3 Department of Pharmacy, The Suqian Clinical College of Xuzhou Medical University, Suqian, China; 4 Department of Pharmacy, Xuzhou Central Hospital, Xuzhou, China

**Keywords:** aplastic anemia, correlation analysis, cyclosporine A, pediatric, trough concentration

**Corresponding author**

Adding/removing a corresponding author

In the published version of this article, Dr. Xintong Mao was erroneously designated as a corresponding author, and Dr. Ling Liu was erroneously omitted as a corresponding author. The correct corresponding authors are Dr. Yi Yun and Dr. Ling Liu.

The original version of this article has been updated.

